# Supplementary material for: Yersinia pseudotuberculosis YopE prevents uptake by M cells and instigates M cell extrusion in human ileal enteroid-derived monolayers
Source: Gut Microbes. 2021 Nov 18;13(1):1988390. doi: 10.1080/19490976.2021.1988390 (PMC8604394; doi:10.1080/19490976.2021.1988390)
Supplement: Supplemental Material [file KGMI_A_1988390_SM8797.pdf]

Supplemental Information

Supplemental Table 1. Table of significances for Figure 4K.[a]

	0 *Yptb* Internalized								
	WT 37°C	Δ*yscNU*	WT 26°C	Δ*yopO*	Δ*yopH*	Δ*yopE*	*yopE*rec	Δ*yopEO*	Δ*yopEH*
WT 37°C									
Δ*yscNU*	****								
WT 26°C	****	ns							
Δ*yopO*	ns	****	****						
Δ*yopH*	ns	****	****	ns					
Δ*yopE*	****	ns	ns	****	**				
*yopE*rec	ns	****	****	ns	ns	****			
Δ*yopEO*	****	ns	ns	****	**	ns	****		
Δ*yopEH*	****	ns	ns	****	****	ns	****	ns	

	1-9 *Yptb* Internalized								
	WT 37°C	Δ*yscNU*	WT 26°C	Δ*yopO*	Δ*yopH*	Δ*yopE*	*yopE*rec	Δ*yopEO*	Δ*yopEH*
WT 37°C									
Δ*yscNU*	ns								
WT 26°C	ns	ns							
Δ*yopO*	ns	ns	ns						
Δ*yopH*	ns	ns	*	*					
Δ*yopE*	ns	ns	*	ns	ns				
*yopE*rec	ns	ns	ns	ns	ns	ns			
Δ*yopEO*	****	****	****	****	**	**	***		
Δ*yopEH*	ns	ns	ns	ns	ns	ns	ns	****	

	10+ *Yptb* Internalized								
	WT 37°C	Δ*yscNU*	WT 26°C	Δ*yopO*	Δ*yopH*	Δ*yopE*	*yopE*rec	Δ*yopEO*	Δ*yopEH*
WT 37°C									
Δ*yscNU*	****								
WT 26°C	****	ns							
Δ*yopO*	ns	****	****						
Δ*yopH*	ns	****	****	ns					
Δ*yopE*	****	****	****	**	**				
*yopE*rec	ns	****	****	ns	ns	***			
Δ*yopEO*	ns	****	****	ns	ns	**	ns		
Δ*yopEH*	****	ns	ns	****	****	***	****	****	

[a] * $p < 0.05$; ** $p < 0.01$; *** $p < 0.001$; **** $p < 0.0001$; ns = no significance

Supplemental Table 2. Additional details related to quantification of Zombie Live/Dead analysis

	Uninfected Monolayers		
	Avg % of Zombie$^+$ Extruding dead cells	Range of mCherry intensity amongst Zombie$^+$ Extruding dead cells	Geometric Mean of mCherry Intensity amongst Zombie$^+$ Extruding dead cells
Expt 1	0.4%	$6x10^6$-$2x10^8$	$7x10^7$
Expt 2	0.4%	$6x10^6$-$1x10^8$	$3x10^7$
Expt 3	0.04%	$5x10^6$-$6x10^7$	$2x10^7$

	Uninfected Monolayers		
	Avg % Zombie$^+$ Non-extruding Cells	Range of mCherry Intensity amongst Zombie$^+$ Non-extruding cells	Geometric Mean of mCherry Intensity amongst Zombie$^+$ Non-extruding cells
Expt 1	3.1%	$4x10^4$-$9x10^7$	$5x10^5$
Expt 2	1.0%	$9x10^4$-$9x10^6$	$5x10^5$
Expt 3	0.4%	$1x10^5$-$8x10^5$	$4x10^5$

	# M cells analyzed per experiment		
	UN ([a,b])	WT 37°C ([a,b])	Δ*yscNU* ([a,b])
Expt 1	29 (0,2)	6 (1,2)	6 (0,3)
Expt 2	ND[c]	18 (2,5)	14 (0,2)
Expt 3	8 (0,0)	8 (0,3)	7 (0,0)

[a] Number of Zombie+ cells in intensity range of Extruding Dead Cells
[b] Number of Zombie+ cells in intensity range of Non-extruding cells
[c] ND = Not determined

Supplemental Table 3. Additional details related to quantification of cleaved caspase 3 analysis

	Uninfected Monolayers		
	Avg % of CC3$^+$ cells	Range of mCherry Intensity amongst CC3$^+$ cells	Geometric Mean of mCherry Intensity amongst CC3$^+$ cells
Expt 1	1.5%	$1x10^5$-$1x10^6$	$3x10^5$
Expt 2	2.2%	$1x10^5$-$5x10^6$	$4x10^5$
Expt 3	5.1%	$1x10^5$-$3x10^7$	$4x10^5$

	WT 37°C-Infected Monolayers	
	Avg % of CC3$^+$ cells	# M cells analyzed per expt ([a])
Expt 1	0.2%	10 (1)
Expt 2	0.8%	10 (0)
Expt 3	5.1%	20 (3)

[a] Number CC3+ M cells per experiment

Supplemental Table 4. Table of significances for Figure 6J.[a]

	0 *Yptb* Internalized				
	WT 37°C	Δ*yopE*	*yopER144A*	*yopESptP*	*yopEL109A*
WT 37°C					
Δ*yopE*	****				
yopER144A	****	ns			
yopESptP	ns	**	**		
yopEL109A	ns	****	****	ns	

	1-9 *Yptb* Internalized				
	WT 37°C	Δ*yopE*	*yopER144A*	*yopESptP*	*yopEL109A*
WT 37°C					
Δ*yopE*	ns				
yopER144A	ns	ns			
yopESptP	ns	ns	ns		
yopEL109A	ns	ns	ns	ns	

	10+ *Yptb* Internalized				
	WT 37°C	Δ*yopE*	*yopER144A*	*yopESptP*	*yopEL109A*
WT 37°C					
Δ*yopE*	***				
yopER144A	****	ns			
yopESptP	ns	ns	ns		
yopEL109A	ns	**	***	ns	

[a] ** $p < 0.01$; *** $p < 0.001$; **** $p < 0.0001$; ns = no significance

Supplemental Table 5. *Yersinia* strains used in this study.

Strain Number	Description	Original Strain #	Reference for original strain
ACF053	*Yptb* YPIII pIB1, pACYC184-ptet::GFP	JM301	[1]
ACF054	*Yptb* YPIII pIB1, Δ*yadA* + pACYC184-ptet::GFP	MM92	this study
ACF055	*Yptb* YPIII pIB1, Δ*inv* + pACYC184-ptet::GFP	ERG9	this study
ACF056	*Yptb* YPIII pIB1, Δ*inv/yadA* + pACYC184-ptet::GFP	FM420	this study
ACF057	*Yptb* YPIII pIB1, YopETEM	FM324	[2]
ACF068	*Yptb* YPIII pIB1, Δ*yopE/sycE*+pACYC184-ptet::GFP	LL31	[1]
ACF073	*Yptb* YPIII pIB1, Δ*yscN-U* +pACYC184-ptet::GFP	JMB31	[3]
ACF074	*Yptb* YPIII pIB1	JM301	[1]
ACF076	*Yptb* YPIII pIB1, pMMB67EH-ptetA::mCherry	JM301	[1]
ACF085	*Yptb* YPIII pIB1, Δ*yopE* + YopEL109A +pACYC184-GFP	WS315a	[4]
ACF087	*Yptb* YPIII pIB1, Δ*yopE* + YopER144A + pACYC184-GFP	WS312	[4]
ACF091	*Yptb* YPIII pIB1, Δ*yopE* + YopE1-100SptP166-293 + pACYC184-ptet::GFP	WS352	[4]
ACF093	*Yptb* YPIII pIB1, Δ*yopE* + YopE recombined +pACYC184-ptet::GFP	WS338	[4]
ACF112	*Yptb* YPIII pIB1, Δ*yopH* + pACYC184-ptet::GFP	this study	this study
ACF113	*Yptb* YPIII pIB1, Δ*yopEH* + pACYC184-ptet::GFP	this study	this study
ACF114	*Yptb* YPIII pIB1, Δ*yopO* + pACYC184-ptet::GFP	this study	this study
ACF115	*Yptb* YPIII pIB1, Δ*yopEO* + pACYC184-ptet::GFP	this study	this study

References for Supplemental Information

1. Logsdon, L.K. and J. Mecsas, *Requirement of the Yersinia pseudotuberculosis effectors YopH and YopE in colonization and persistence in intestinal and lymph tissues.* Infect Immun, 2003. **71**(8): p. 4595-607.
2. Maldonado-Arocho, F.J., et al., *Adhesins and host serum factors drive Yop translocation by yersinia into professional phagocytes during animal infection.* PLoS Pathog, 2013. **9**(6): p. e1003415.
3. Balada-Llasat, J.M. and J. Mecsas, *Yersinia has a tropism for B and T cell zones of lymph nodes that is independent of the type III secretion system.* PLoS Pathog, 2006. **2**(9): p. e86.
4. Songsungthong, W., et al., *ROS-inhibitory activity of YopE is required for full virulence of Yersinia in mice.* Cell Microbiol, 2010. **12**(7): p. 988-1001.
